# Clinical science, responsibilities and society

**DOI:** 10.1007/s12471-020-01377-2

**Published:** 2020-02-25

**Authors:** J. M. Ribeiro, P. P. T. de Jaegere

**Affiliations:** 1 Department of Cardiology, Thoraxcenter, Erasmus Medical Centre, Rotterdam, The Netherlands; 2 Department of Cardiology, Centro Hospitalar e Universitário de Coimbra, Coimbra, Portugal

Max Planck proclaimed that ‘science cannot solve the ultimate mystery of nature …’ and that was—in his opinion—since ‘we are part of the mystery we try to solve’. Most will agree with the first part of the statement; the question is what he meant with the second. The problem with the ‘nature’ that is the subject of clinical research is that it is quite complex and that we have limited technical tools and understanding to grasp that convoluted reality.

The study by van Lavieren et al. is an illustration thereof [1]. The relationship between admission glucose level in non-diabetic patients with a first ST-segment elevation myocardial infarction (STEMI) and microvascular function of the infarct-related (IRA) and non-infarct (reference) artery was studied. For that purpose, coronary flow velocity reserve (CFVR) was calculated of the IRA and reference vessel, of which the baseline (BMR) and hyperaemic (HMR) microvascular resistance was also calculated provided the percentage diameter stenosis was <30%. The article by van Lavieren et al. concerns an observational study with its inherent and acknowledged limitations (e.g. sample size, inclusion and exclusion criteria, incomplete follow-up, glucose level measured only once at admission without further biochemical details of glucose metabolism) using unadjusted and adjusted linear regression. The main finding was an absence of such an association in the IRA but a lower CFVR with increasing glucose level in the reference vessel plausibly due to a combination of a higher flow and lower resistance at baseline.

In other words, while during acute percutaneous coronary intervention (PCI) all our attention is focused on the IRA, microvascular dysfunction occurs at remote sites of the heart that may need our attention since there may be prognostic implications. This endeavour must be applauded, as it concerns a study of complex methodology that adds insight into a poorly known pathophysiological process and thus may help to propose adjunctive measures to improve the efficacy of treatment.

Biological processes, however, are multidimensional and thus linear regression may fail to elucidate the complex associations of factors that play a role in outcome (i.e. microvascular function). This is not only a sample size issue (see regression line and slope in the scatter plot) but more importantly that relationships in biological processes may be non-linear. Also, the nature of the association remains unclear (cause-and-effect, indirect, epiphenomenon, etc.). Albeit subject to debate, factors other than secondary acute stress-related biochemical factors may play a role as well as indicated by the multivariate regression (heart rate, changes in regional myocardial wall tension). Interestingly, in an attempt to elucidate the pathophysiology of microvascular (dys)function during STEMI, the answer to the original hypothesis generates novel hypotheses. Instead of focussing on biochemical reactions and consequently counteracting these (e.g. by GIK administration) unloading of the myocardium may eventually be more beneficial [2].

Tijssen et al. report the circa 4‑year outcomes of 135 patients enrolled in the AMC Absorb Registry (2012–2013) who were treated with a bioresorbable vascular scaffold (BVS) for stable or unstable coronary artery disease (both roughly 50%) [3]. The authors postulate real-world practice but selection bias cannot be ruled out since the decision to implant a BVS was left to the discretion of the operator. Also, the total number of patients treated with PCI during that period is not mentioned. The 4‑year cumulative cardiac death rate, target-vessel myocardial infarction and target-vessel revascularisation (TVR) were 3.2%, 6.9% and 16.7%, respectively. Target lesion revascularisation (included in or excluded from TVR?) was 14.6% and the incidence of ‘definite’ scaffold thrombosis was 3.8% (one case at 911 days in a patient with stable angina and type A lesion). Given that follow-up was by telephone contact and status examination, these point estimates—except for death—likely represent an underestimation of their true value. More importantly, the Kaplan-Meier curve reveals a continuous occurrence during the entire follow-up period, albeit most cases occur within 18 months after BVS implantation. The authors rightfully conclude that events ‘continue to accrue beyond two years’.

There is little discussion that, when PCI is indicated, the ideal treatment consists of the implantation of a non-permanent device (scaffold). Yet, given the findings of Tijssen et al. and those of randomised controlled trials, one may question whether the technology is ready for use notwithstanding its approval (EU, US authorities). As suggested by the authors, procedure-/operator-related factors may play a role. The role of proper sizing (plus length) and implantation technique was shown soon after the introduction of the permanent scaffold (stent) [4]. Yet, outcome is also determined by patient-related variables as indicated by the ABSORB II and III trials (i.e. lower event rate when excluding patients with acute coronary syndrome and complex lesions) and device-related factors [5, 6]. The implantation of a BVS is invariably associated with reactive (e.g. inflammatory) vessel wall responses that are still incompletely understood in terms of nature and time course. It, thus, appears that BVS technology should only be used in clinical trials with strict and independent analysis and surveillance.

Pustjens et al. have published an elegant position paper concerning the management of patients with acute myocardial infarction (MI) and non-obstructive coronary artery disease (MINOCA) [7]. The article concerns a difficult clinical entity given that the definition of MI is subject to interpretation and given the many cardiac and non-cardiac conditions possibly associated with MINOCA. Some can be diagnosed easily and accurately, others cannot (most often cardiac conditions) due to technical issues and issues of sensitivity, specificity, etc. In addition, the causative relationship between finding and clinical presentation may be doubtful. The authors provide a well-designed diagnostic algorithm stressing that it concerns a dynamic working diagnosis. This paper nicely illustrates the complexity of the mystery that we try to solve, as well as the limited insight we still have despite the many technical tools.

Zwart et al. published an interesting paper on the management of patients with non-ST-segment elevation acute coronary syndromes (NSTE-ACS) as they draw our attention to which patients are truly at high risk and merit early/urgent revascularisation (GRACE >140) and by fine-tuning the ESC guidelines to the Dutch context with its typical geography, high level of care and organisation [8]. Most patients with NSTE-ACS are indeed not truly at high risk and, therefore, same-day transfer to a PCI centre can be avoided. In these patients further and appropriate treatment decisions can be taken by the heart team incorporating relevant patient-related factors (e.g. age, antecedents, co-morbidity, etc.) in combination with the coronary anatomy.

Consequently, the physician and sound clinical sense are reinstated as the imperative safeguard of the appropriateness of care. What is needed, however, is a reassessment of how the organisation of care in the Netherlands needs to be adapted to these insights. It can be argued that organisational efficiency has become the main driver of the execution of care (i.e. quick turn-over) rather than the selection of treatment and timing that best fits the individual patient. This stems from the policy during the 1990s by which medicine came to be considered a commercial product in combination with cost containment, whereby the replacement of hospitals by medical centres was in part followed by a reduction in the numbers of paramedics and beds. The key word is time. Time is money, the more time we take (back) as professionals, the more appropriate and, conceivably, more cost-effective will be the delivery of care.

The beauty of peer-reviewed journals such as the *Netherlands Heart Journal* is that after revision and more distant reflection, as yet unnoticed concepts may emerge, stimulating further thinking and research in our quest to ‘solve the mystery of nature’ and to deliver care as best we can, whereby hospital management and authorities have a responsibility as well.
